# Supplementary material for: Large language models can consistently generate high-quality content for election disinformation operations
Source: PLoS One. 2025 Mar 17;20(3):e0317421. doi: 10.1371/journal.pone.0317421 (PMC11913289; doi:10.1371/journal.pone.0317421)
Supplement: S3 Table — (PDF) [file pone.0317421.s003.pdf]

S3 Table. Information sheet shown to experiment participants.

**Participant Information Sheet**
You will see several pieces of social media content which contain misinformation about an anonymous MP from the UK misusing campaign funds. On current social media, content is sometimes generated by AI, or 'bots', designed to look like human-made content. Our study is designed to reflect this, with different content presented randomly for each participant, so that some participants may see content that has been generated by AI alongside the human-made content. Your task is to indicate whether you think each piece of content is written by AI or humans.<br>We will also ask for some demographic information such as age, gender, education level and political orientation. Before the main task, we will also ask some short questions about how familiar you are with digital environments. To take part you must be 18 or over, fluent in English and resident in the UK.<br>Please note that the items you will read contain misinformation (that is, information which is not true) about an anonymous UK MP. You should not take part if you feel completing the survey may adversely affect your emotional state in any way. If you choose to take part, you are free to withdraw at any time and without giving a reason. If you decide to withdraw, you can close the page and your data will not be used. All of the information you provide will remain anonymous. If you decide to take part, you will be asked to electronically sign a consent form after reading this information sheet.You will then be taken through the study.
