# Supplementary material for: Large language models can consistently generate high-quality content for election disinformation operations
Source: PLoS One. 2025 Mar 17;20(3):e0317421. doi: 10.1371/journal.pone.0317421 (PMC11913289; doi:10.1371/journal.pone.0317421)
Supplement: S4 Table — (PDF) [file pone.0317421.s004.pdf]

S4 Table. Demographic statistics of experiment participants.

Attribute	Value	exp_MP$_L$	exp_MP$_R$	exp_VT
Age	18-24	10.4%	9.0%	13.8%
	25-34	24.7%	26.8%	35.4%
	35-44	21.8%	27.2%	26.7%
	45-54	21.4%	17.2%	14.0%
	55-64	14.2%	12.6%	7.3%
	65+	7.4%	7.2%	2.9%
Gender	Female	53.8%	56.6%	56.9%
	Male	44.2%	42.0%	41.7%
	Non-binary / 3rd gender	1.3%	0.8%	0.9%
	Prefer not to say	0.1%	0.3%	0.4%
	Prefer to self-describe	0.5%	0.4%	0.1%
Highest Education Level	Graduate Degree	17.9%	19.3%	29.7%
	Bachelors Degree	41.0%	40.7%	44.9%
	Vocational	13.7%	13.7%	7.1%
	Some university	8.1%	7.3%	9.8%
	Completed Secondary School	18.1%	18.4%	7.5%
	Some Secondary School	1.0%	0.5%	0.6%
	Completed Primary School	0.0%	0.0%	0.3%
	Prefer not to say	0.1%	0.1%	0.1%
Political Affiliation	Strong LW (> 50%)	31.8%	33.0%	29.9%
	Mild LW (<= 50%)	20.1%	18.7%	45.3%
	Mild RW (<= 50%)	30.8%	29.3%	20.0%
	Strong RW (> 50%)	17.2%	19.0%	4.6%
	Prefer not to say	0.1%	0.0%	0.1%
Tech Interest	High (> 75%)	40.4%	43.1%	46.1%
	Moderate (> 50%)	41.9%	40.4%	39.9%
	Mild (> 25%)	13.1%	12.1%	10.8%
	Low (<= 25%)	4.6%	4.4%	2.0%
	Prefer not to say	0.0%	0.0%	1.3%
Tech Informed	High (> 75%)	20.9%	22.6%	24.0%
	Moderate (> 50%)	43.5%	44.5%	48.6%
	Mild (> 25%)	28.2%	25.5%	21.2%
	Low (<= 25%)	7.4%	7.3%	5.0%
	Prefer not to say	0.0%	0.0%	1.3%
