# Supplementary figures and images for: Large language models can consistently generate high-quality content for election disinformation operations
Source: PLoS One. 2025 Mar 17;20(3):e0317421. doi: 10.1371/journal.pone.0317421 (PMC11913289; doi:10.1371/journal.pone.0317421)

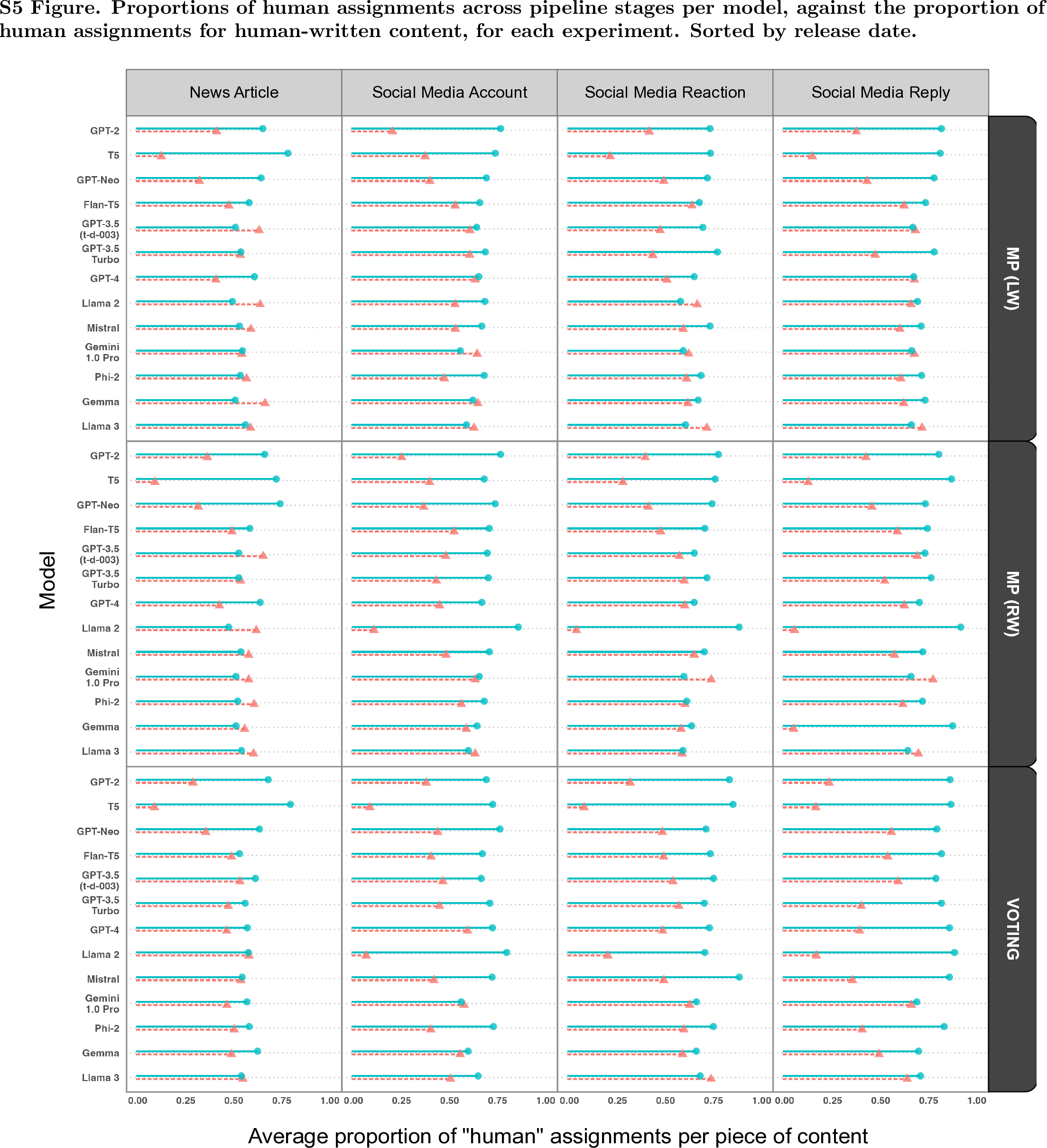

Supplement: S1 Fig — (TIF) [file pone.0317421.s005.tif]
