# Supplementary material for: Large language models can consistently generate high-quality content for election disinformation operations
Source: PLoS One. 2025 Mar 17;20(3):e0317421. doi: 10.1371/journal.pone.0317421 (PMC11913289; doi:10.1371/journal.pone.0317421)
Supplement: Attachment — Submitted filename: PLOS ONE Rebuttal Letter.docx [file pone.0317421.s007.docx]

Dear Editor,

We thank the reviewers for their generous comments, and the editor for their work to consolidate this feedback. We have addressed the issues raised below, and believe the manuscript has been significantly improved by the changes. We remain at your disposal for any further modifications required.

Original reviewer comments are presented in *italic blue.*

**Reviewer 1:**

*Typo highlighted on page 5: “Weidinger et al. [25] propose a sociotechnical approach these safety evaluations, consisting of evaluations at three intersecting levels”*

Thank you, we have added “to” in between “approach” and “these”.

*Page 8: Inconsistent use of hyphens in the sentence discussing UK context*

Thank you, we have addressed this.

*Page 15: The sentence about GPT-3.5 Turbo and Mistral's refusal patterns needs clarification. Consider rewording to better explain the relationship between refusals in DisElect.BL versus DisElect.VT/MP*

We appreciate this discussion may not have been sufficient, and have expanded on this paragraph to discuss what refusals on Diselect.BL represent.

*Fig.1: The ordering principle for LLMs in the visualization is not immediately clear. Consider explicitly stating the ordering criteria or reorganizing based on a clear principle (e.g., chronological order or by company)*

Thanks for your concern, we appreciate that the figures and their captions are not displayed together in the materials provided to reviewers so it was a bit hard to grasp this! To help address this, we have included a comment at the beginning of the results section to make clear that models are sorted by release date in all visualisations, as well as emphasising this in the caption. .

*The paper mentions the release date of LLMs multiple times and finds it correlated with the humanness. However, there is no specific presentation of this. Consider adding a timeline figure or table showing this.*

That’s a good idea – we have added Fig. 8 to visualise this.

*Page 19: The discussion of GPT-3.5 Turbo's consistent performance across stages (std = .03) would be more compelling with a supplementary figure showing mean and standard deviation of performance across stages for all LLMs*

Thank you, this makes sense – We have added Fig. 7 to explicitly visualise standard deviation (both across stages and experiments), and have expanded the discussion on page 19 to accompany this.

*Page 23: Typographical error "LLms" should be "LLMs"*

Thank you, we have fixed this.

*Page 24: Abbreviation "OR" is used before being defined as "Odds Ratio"*

Thank you for spotting this, we have addressed this.

**___**

**Reviewer 2:**

*While the authors acknowledge the limited number of LLMs evaluated, a more thorough discussion on what the difefrent outcomes of model actually mean would be a goo addition. Since that might point towards training data biasness.*

Thank you, we agree that understanding the biases of models encoded through training data is an important aspect of the explainability of the results of these models. We have expanded the results section for the DisElect eval, further developing the discussion on our analysis of how refusal rates vary across different parameters such as MP gender and party to link these findings to potential representation imbalances in training data to address this (see page 18).

*Further investigation into the impact of prompt engineering and red-teaming on LLM compliance and humanness perception would provide a more complete understanding of the potential for malicious use. This I beleive will be very critical in future usage of these kind of methods*

It’s a great point that adversarial prompting is inevitable in malicious use - we think that that our study shows that on this kind of task malicious actors can generate high-impact content without need for any adversarial strategies (because most models don’t refuse, and achieve high-humanness as is), and the work/cost involved for a malicious actor in deploying them is lower without adversarial strategies. We have added a section on adversarial prompting to our literature review (see page 6), and have made more explicit references to and discussions of adversarial strategies throughout this paper to try to address this (page 28, 29, 30).

As you suggest, the potential for using prompt engineering to generate responses that score more highly in humanness is great idea that we think would be a really interesting aspect of future work exploring how humans perceive AI generated content, and have made reference to this in the future work section (page 30).

*I have some reservations of using LLM as judge method. Specifically for these kind of study, it would also mean we are trusting another LLM to validate a "perception" of biasness form another LLMs output. A human evaluation element i believe not only strengthens the paper but might be necessary. I'll be very happy to see such an element added and if that affects the papers conclusions.*

Propagating bias by using models to evaluate other models is definitely a fundamental concern that we share, in the risks this poses to scientific validity and false perceptions of the safety and fairness of AI models, and we appreciate you raising it.

We do include a human evaluation of our LLM judge approach, presenting performance metrics on a human-annotated sample of model responses. We have expanded our discussion of LLM-as-a-judge as a method for evaluating other LLMs, as it was perhaps not sufficiently well signposted in the first draft (see page 12/13). Our analysis shows that identifying clear patterns or semantics in text, such as whether a model response is refusing to comply with an instruction, seems to be a task that is well suited to an LLM, which is why we deploy it for this part of our study.

We completely agree that more complex, subjective judgements, where bias may play a more significant impact, are much harder to justify using an LLM to evaluate. As such, the latter half of the paper focuses exclusively on human evaluation of LLM outputs to explore how humans perceive this content compared to human-written content, precisely because we believe that evaluating the safety of AI models requires nuanced human perspectives and judgements - we also study the biases of the human participants themselves in this element.

___

We hope the revisions and explanations made in this letter are sufficient for the approval of this study for publication.

Kind Regards,

**Angus R Williams**

Senior Data Scientist

On behalf of all authors.
